# GRK5 is required for adipocyte differentiation through ERK activation

**DOI:** 10.21203/rs.3.rs-4360297/v1

**Published:** 2024-05-17

**Authors:** Chia-Chi Chuang Key, Mary Seramur, Bailey McDonald, Matthew Davis Davis, Leah Solberg Woods

**Affiliations:** Wake Forest University School of Medicine; Wake Forest University School of Medicine; Wake Forest University School of Medicine; Wake Forest School of Medicine; Wake Forest School of Medicine

## Abstract

Previous studies have identified G protein-coupled receptor (GPCR) kinase 5 (GRK5) as a genetic factor contributing to obesity pathogenesis, but the underlying mechanism remains unclear. We demonstrate here that Grk5 mRNA is more abundant in stromal vascular fractions of mouse white adipose tissue, the fraction that contains adipose progenitor cells, or committed pre-adipocytes, than in adipocyte fractions. Thus, we generated a GRK5 knockout (KO) 3T3-L1 pre-adipocyte to further investigate the mechanistic role of GRK5 in regulating adipocyte differentiation. During adipogenic stimulation, GRK5 KO pre-adipocytes were unable to achieve mature adipocyte morphology and lipid accumulation compared to wildtype cells coupled with suppressed adipogenic and lipogenic gene expression. Beside GPCR signaling, RNA sequencing and pathway analysis identified insulin-like growth factor 1 (IGF-1) signaling to be one of the top 5 significantly dysregulated pathways in GRK5 KO cells. GRK5 KO cells also displayed decreased insulin-stimulated ERK phosphorylation, a downstream target of insulin-stimulated IGF-1 receptor activation, suggesting that GRK5 acts through this critical pathway to impact 3T3-L1 adipocyte differentiation. To find a more translational approach, we identified a new small molecule GRK5 inhibitor that was able to reduce 3T3-L1 adipogenesis. These data suggest that GRK5 is required for adipocyte differentiation through IGF-1 receptor/ERK activation and may be a promising translational target for obesity.

## Introduction

Prevalence of obesity continues to increase, along with related comorbidities (1, 2, 3). Adipose tissue plays a central role in obesity resulting in adipocyte hyperplasia (increased adipocyte number) and/or adipocyte hypertrophy (increased adipocyte size), although the underlying causes of adipose tissue expansion are generally unknown. Our previous work identified G protein-coupled receptor (GPCR) kinase 5 (GRK5) as a candidate causal gene for visceral adiposity, where *Grk5* expression positively correlated with retroperitoneal and epididymal white fat pad mass in rats (4) and *Grk5* knock-down in 3T3L1 pre-adipocytes led to decreased total triacylglycerol (TAG) accumulation in mature adipocytes (4). In humans, GRK5 gene expression in white subcutaneous white adipose tissue is positively correlated with BMI (kg/m^2^) in the African American Genetics of Metabolism and Expression (AAGMEx) cohort (r = 0.22, p = 0.0003), a group of 256 African Americans with in depth glucometabolic phenotyping and adipose tissue transcriptome analysis (5). In addition, Wang et al. reported that whole-body GRK5 knockout (KO) mouse model demonstrated protection against diet-induced obesity along with decreased adipogenesis compared to wildtype (WT) control mice (6). These findings indicate that GRK5 is likely a causal gene for adiposity and may serve as a target to treat obesity. However, the mechanisms by which GRK5 contribute to adiposity and obesity remain unclear.

The GRK family consists of 7 proteins (GRK1–7) that can terminate GPCR signaling through phosphorylation of cognate receptors, resulting in recruitment of β-arrestin, which then facilitates receptor internalization and degradation (7). The GTEx RNA sequencing dataset showed that subcutaneous white adipose tissue demonstrates the second highest GRK5 mRNA expression, with only lung being higher, in humans (8). In mice, GRK5 mRNA tissue distribution is similar to that of humans (i.e., highest in the lung, followed by subcutaneous white fat)(9). Although GRK5 is highly expressed in adipose tissue (8, 9), the Human Protein Atlas reported that GRK5 is present in endothelial cells and adipose progenitor cells (committed pre-adipocytes), but not in mature adipocytes, of human white visceral and subcutaneous fat pads (10). This is in agreement with a recent single cell/nuclei RNA sequencing analysis of human and mouse white adipose tissue that revealed virtually no GRK5 transcripts in mature adipocytes (11). Thus, we have created a novel GRK5 KO 3T3-L1 pre-adipocyte cell line to investigate the role of GRK5 in the regulation of adipocyte differentiation and function.

3T3-L1 pre-adipocytes are differentiated into mature insulin-responsive adipocytes by exposing a quiescent population of confluent cells to a classical adipogenic cocktail, including 3-isobutyl-1-methylxanthine (IBMX), dexamethasone (DEX), and insulin, that activates a cascade of transcription factors. IBMX is used to increase cAMP levels to activate cAMP responsive-element binding protein (CREB) (12). When activated, CREB translocates to the nucleus and binds to CRE to promote expression of CCAAT/enhancer-binding protein (C/EBP) and peroxisome proliferator-activated receptor gamma (PPARγ) (13, 14). DEX is used to directly induce C/EBP and PPARγ activity (15, 16). C/EBP and PPARγ coordinate the expression of numerous genes such as fatty acid synthase (FASN) for *de novo* fatty acid synthesis, acyl-CoA: diacylglycerol acyltransferase (DGAT) for fatty acid esterification, and cluster of differentiation 36 (CD36) for fatty acid transport, all of which are associated with the mature adipocyte phenotype.

Insulin, acting through the insulin-like growth factor-1 (IGF-1) receptor, a receptor tyrosine kinase, is also required to ensure complete conversion of 3T3-L1 pre-adipocytes into mature adipocytes (17). Recently, the Farmer group demonstrated that insulin induces a robust transient activation of the extracellular signal-regulated kinase (ERK) pathway during the first 12 hours of 3T3-L1 adipogenesis, and that this is required for subsequent adipocyte differentiation by activating C/EBP and PPARγ (18). Insulin/IGF-1 receptor and ERK can communicate in several ways to activate adipocyte differentiation. In this study, we will explore a novel hypothesis that GRK5 is involved in adipocyte differentiation by regulating the insulin/IGF-1 receptor/ERK pathway suggesting that GRK5 is not only a GPCR kinase, but also governs receptor tyrosine kinase signaling in pre-adipocytes to control adipogenesis.

## Materials and Methods

### Mice

Mice were housed in standard cages under a 12-h light cycle and 12-h dark cycle (dark from 6:00 PM to 6:00 AM) at standard ambient temperature and humidity conditions and were provided with ad libitum water and a standard chow diet (Purina-LabDiet, Prolab RMH 3000). All experiments were performed using a protocol approved by the Institutional Animal Care and Use Committee at Wake Forest University School of Medicine in facilities approved by the American Association for Accreditation of Laboratory Animal Care.

To assess the impact of high fat diet on *Grk5* expression, 8-week-old male C57BL/6J mice (Jackson Lab, Bar Harbor, MN, USA, Strain #000664) were fed chow or a high fat diet (Envigo Indianapolis, IN, USA, #TD 88137, 42% from fat, 0.2% total cholesterol) for 16 weeks. Mice were fasted overnight before being euthanized, and adipose tissue (including visceral, subcutaneous, and brown) were collected and stored at − 80°C until used for gene expression. To determine where in adipose tissue Grk5 is expressed, 6-week-old male C57BL/6J mice were fed chow or a high-fat diet (Research Diets Inc, New Brunswick, NJ, USA, #D12492, 60% from fat) for 12 weeks. After 10 weeks on diet, mice went through EchoMRI^™^ analysis, which measure body composition (e.g., fat mass) of live mice. After 12 weeks on diet, mice were fasted for 16 hours and epidydimal visceral white fat pads were harvested and used for adipose tissue digestion (19). Brie y, tissue was enzymatically digested in a digestion buffer (0.5 g of fat in 10 ml) containing 0.8 mg/ml of collagenase II (Worthington Biochemical Corp., Lakewood, NJ, USA), 3% of fatty acid free-BSA (Sigma-Aldrich, Burlington, MA, USA), 1.2 mM of calcium chloride (Sigma-Aldrich), 1 mM of magnesium chloride (Sigma-Aldrich), and 0.8 mM of zinc Chloride (Sigma-Aldrich) in Hanks Buffered Salt Solution (Life Technologies, Carlsbad, CA, USA) for 60 min in a shaking water bath at 37°C with 200 rpm agitation. The fat digest was then filtered through a 200-um filter (Fisher Scientific, Pittsburgh, PA, USA). The adipocyte fraction and stromal vascular (SV) fraction were collected by centrifugation at 800 *g* for 10 min. Red blood cells in the SV fraction were lysed using ACK lysis buffer. The adipocyte and SV fractions were treated with QIAzol Lysis Reagent (Qiagen, Venlo, Netherlands) and stored at − 80°C until used for gene expression.

### Cell cultures

The 3T3-L1 pre-adipocyte cell line was purchased from ATCC (CL-173^™^). The GRK5 KO 3T3-L1 pre-adipocyte was generated using CRISPR gene editing and provided by Synthego Corporation, Redwood City, CA (cells are available upon request). The guide sequence (i.e., TATGTGACAAGCAACCAATT) was designed to target exon 3 of *Grk5* (**Supplementary Fig. 1A**). The KO clone was cut and had a nucleotide removed (i.e., A) during the non-homologous end joining repair process, resulting in a frameshift mutation that causes premature termination of translation at a new nonsense codon, as confirmed by the Sanger sequence (**Supplementary Fig. 1A**).

GRK5 KO 3T3-L1 and its wildtype (WT) control 3T3-L1 pre-adipocytes were first used to assess their proliferation rate using Click-iT^®^ EdU cell proliferation kit (ThermoFisher Scientific, Waltham, MA, USA) based on the manufacture’s procedure. Brie y, cells were seeded into a 6-well plate with a density of 0.1×10^6^ per well in Dulbecco’s Modified Eagle Medium (DMEM, Gibco, Billings, MT, USA) supplemented with 10% iron-fortified calf serum (CS, Sigma-Aldrich) and 1% penicillin/streptomycin (P/S, Gibco) for 24 hours. Cells were then treated with EdU (5-ethynyl-2’-deoxyuridine) solution and incubated for 24 hours. EdU, a nucleoside analog of thymidine, was incorporated into newly synthesized DNA and fluorescently labeled with a bright, photostable Alexa Fluor^™^ 647 dye. Total DNA was stained using Hoechst 3342 and imaged using BioRad ZOE Fluorescent Cell Imaging System.

GRK5 KO 3T3-L1 and WT control 3T3-L1 pre-adipocytes were cultured and differentiated into adipocytes as described previously (19). Briefly, pre-adipocytes were seeded at 0.05 × 10^6^ cells per well in a 6-well culture plate. Cells were cultured in DMEM supplemented with 10% iron-fortified CS and 1% P/S for 48 h until ~90% cell confluence. Adipogenesis (Day 0) was induced by changing the medium to DMEM containing 10% fetal bovine serum (FBS, Sigma-Aldrich) plus an adipogenic cocktail (Sigma-Aldrich) including 1 μg/ml of insulin, 0.25 μM of dexamethasone, 0.5 mM of 3-isobutyl-1-methylxanthine, and 2 μM of rosiglitazone for 3 days (Day 3). Cells were then treated with 1 μg/ml of insulin only for 3 days (Day 6) and then without any adipogenic reagents for the next 3 days (Day 9). The medium was changed every 2 days. At Days 0, 3, 6 and 9 of differentiation, cells were stained with Oil-Red O and imaged using the BioTek Cytation C10 Confocal Imaging Reader (Agilent BioTek, Winooski, VT, USA) as well as lipid extracted for triacylglycerol (TAG) measurement as previously described (19).

In order to examine ERK expression, Day 2 differentiated WT and GRK5 KO cell cultures were serum starved overnight and then treated with 1 μg/ml of insulin for 5, 10, and 15 minutes. The cellular proteins were harvested as described in the section below for Western blot analysis.

### RNA extraction and real-time PCR

Total RNA was harvested from cells and tissues using QIAzol Lysis Reagent and isolated by following the protocol described in the RNeasy Lipid Tissue Mini Kit (Qiagen). The concentration and quality of RNA were determined using a Nanodrop (ThermoFisher Scientific) and standardized to 1 μg of RNA for cDNA synthesis. The cDNA was prepared with the OmniScript RT Kit (Qiagen) and stored at −20°C until used for real-time PCR. Real-time PCR was performed in duplicate on the QuantStudio^™^ 3 systems (ThermoFisher Scientific) using TaqMan^®^ Fast Advanced Master Mix and TaqMan^®^ gene expression assays (ThermoFisher Scientific) including Grk5 (Mm00517039_m1), *Cd36* (Mm00432403_m1), *Fabp4* (Mm00445878_m1), *Pparγ* (Mm0040940_m1), *Acc1* (Mm01304257_m1), *Fasn* (Mm00662319_m1), *Dgat1* (Mm00515643_m1), *Dgat2* (Mm00499536_m1), *Lipin1* (Mm00550511_m1), and *Lipin2* (Mm00522390_m1) with 18S rRNA (REF 4352655) as a housekeeping gene. Gene expression was normalized to the endogenous control gene 18S rRNA (REF 4352655) and analyzed using the 2ddCt method with 95% confidence.

### Protein extraction and Western blot

Total cellular protein was harvested in Pierce^™^ IP lysis buffer (ThermoFisher Scientific) supplemented with cOmplete^™^ EDTA-free Protease (Sigma-Aldrich) and PhosSTOP^™^ Phosphatase (Sigma-Aldrich) inhibitor tablets and frozen at −20°C until used. Protein samples were normalized to 1 mg of protein, prepared in non-reducing laemmli buffer and DTT, and heated at 95°C for 10 minutes. Protein was loaded and separated on a 4–20% polyacrylamide gel (Bio-Rad Laboratories, Hercules, CA, USA) and transferred to a 0.2 μm nitrocellulose membrane (Bio-Rad). Membranes were blocked in 5% non-fat milk in 1X Tris-buffered saline plus 0.1% Tween (TBST, Bio-Rad) for 2 hours at room temperature. Primary antibodies were diluted in TBST with 1% non-fat dry milk and incubated overnight at 4°C with gentle rocking. Primary antibodies were diluted as followed: GRK5 (Santa Cruz Biotechnology, Santa Cruz, CA, USA) at 1:1000, GAPDH (Santa Cruz Biotechnology) at 1:1000, β-actin (Cell Signaling Technology) at 1:1000, Phospho-p44/42 ERK1/2 (Cell Signaling Technology, Danvers, MA, USA) at 1:1000 and Total p44/42 ERK1/2 (Cell Signaling Technology) at 1:1000. Following overnight incubation, membranes were washed 3 times in TBST for 5 minutes with agitation and incubated with secondary antibody in 5% non-fat milk for 1 hour at room temperature (ThermoFisher Scientific mouse and rabbit secondaries, 1:5000) with gentle rocking. Membranes were washed 3 times in TBST for 5 minutes with agitation. SuperSignal^™^ West Pico PLUS Chemiluminescent Substrate (ThermoFisher Scientific) was added to the membrane prior to imaging using the ChemiDoc Gel Imaging System (Bio-Rad). Protein expression was quantified using Bio-Rad ImageLab software.

### RNA sequencing and pathway analysis

GRK5 KO and WT cells were seeded and proliferated for 48 hours. After 48 hours, cells were treated with the adipogenic cocktail as described above for 6 hours, and RNA was collected and extracted as previously described (19). Total RNA was used to prepare cDNA libraries using the Illumina^®^ TruSeq Stranded Total RNA with Ribo-Zero Gold Preparation kit (Illumina Inc., San Diego, CA, USA). The libraries were pooled and sequenced to an estimated target read depth of 40M single-end 100 bp reads per sample on the Illumina NovaSeq 6000. For all samples, 80% of sequences achieved > Q30 Phred quality scores (FASTQC analysis, Babraham Bioinformatics). Adapter contamination was cleaned with Trimmomatic (20). Reads were aligned to the murine reference genome mm39 using the STAR sequence aligner (21), and gene counts determined using featureCounts software (22). Differentially expressed genes were identified using limma (23). We then used Ingenuity Pathway Analysis (IPA) to identify top up-and down-regulated pathways.

### In vitro GRK5 inhibition assays

A pyridine-based bicyclic compound of small molecule GRK5 inhibitor, GRK5-IN-2 (**Supplementary Fig. 2A**), was purchased from MedChemExpress (HY-136561). The half maximal inhibitory concentration (IC50) of GRK5-IN-2 was determined using the ADP-Glo Kinase Assay (Promega, Madison, WI, USA) according to the manufacturer’s instructions. Briefly, a twofold serial dilution of GRK5-IN-2 was carried out in DMSO, and inhibitors were subsequently diluted into assay buffer to the final required concentrations. Each inhibitor dilution was transferred into a white 96-shallow well plate. GRK5 protein (final concentration at 0.5 mg/mL), ATP (final concentration at 25 μM), and casein (final concentration at 20 mg/mL) as the substrate were added to each well. Reactions were incubated for 120 min at the room temperature. Then, ADP-Glo^™^ Reagent was added each well and incubated at room temperature for 40 minutes to stop the kinase reaction and deplete the unconsumed ATP, leaving only ADP and a very low background of ATP. Kinase Detection Reagent was added and incubated at room temperature for 60 minutes to convert ADP to ATP and to introduce luciferase and luciferin to detect ATP using a plate-reading luminometer.

### Fatty acid uptake and lipogenesis

Fatty acid uptake and incorporation into lipids as well as *de novo* lipogenesis were determined using [^3^H]-oleic acid and [^14^C]-acetic acid, respectively, following the procedure adapted from our previous study (19). Day 3 differentiated WT 3T3-L1 pre-adipocyte cultures were labeled with 0.5 μCi of [1,2-^14^C]-acetic acid (PerkinElmer, Waltham, MA, USA) or 5 μCi of [9,10-^3^H(N)]-oleic acid (PerkinElmer) plus 0.04 mM oleic acid (Sigma-Aldrich) conjugated with 0.01 mM fatty acid free-bovine serum albumin (BSA) of DMEM supplemented with 10% FBS, 1% P/S and 1 μg/ml of insulin for 0 (no radioisotopes), 30, 60 and 120 min. Following radiolabeling, cells were washed with ice-cold DPBS twice and lipid-extracted with hexane:isopropanol (3:2, vol:vol). Lipid classes from standards and cellular lipid extracts were separated by thin layer chromatography using Silica Gel plates and a solvent system containing hexane:diethyl ether:acetic acid (80:20:2, vol:vol:vol). Lipids were visualized by exposure to iodine vapor, and bands corresponding to TAG, free cholesterol (FC), cholesteryl ester (CE), and phospholipid (PL) were scraped and counted using a scintillation counter. After lipid extraction, cell residue was dissolved with 0.1 N of NaOH, and protein concentrations were measured using a Pierce^™^ BCA Protein Assay Kit for protein normalization of data.

### Statistics

Data are presented as mean ± standard error of the mean (SEM). All data points reflect biological replicates. Binary comparisons are performed using two-tailed Student’s t-test. Datasets comparing the effect of a single independent variable on more than two groups are assessed by one-way ANOVA followed by Dunnett’s correction. Datasets containing groups defined by two independent variables (genotype, time) are assessed by two-way ANOVA with Sidak’s correction. Prism 10 software (GraphPad) is used to perform statistical analyses (Statistical significance p < 0.05) and generate graphical representations of data.

## Results

### Grk5 is highly expressed in the stromal vascular fraction of mouse white adipose tissue.

**Figure 1:**
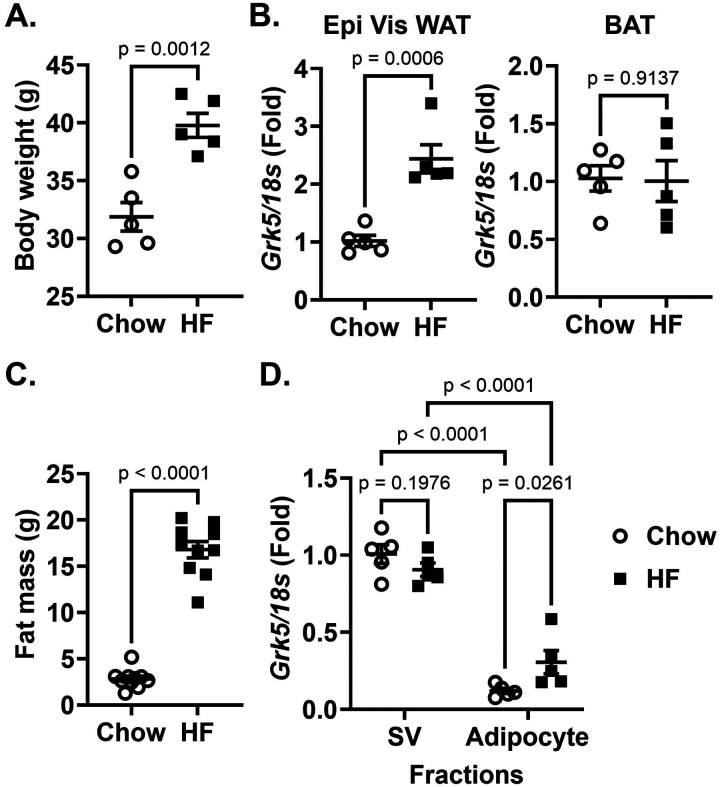
The relationship between Grk5expression and adiposity. **(A)**Eight-week-old male C57BL/6J mice were fed chow or a high fat diet (Envigo #TD 88137, 42% from fat, 0.2% total cholesterol) for 16 weeks and their body weight was measured (n=5/diet group). **(B)** Mice were then fasted for 24 hours and their epididymal (Epi) visceral (Vis) white adipose tissue (WAT) and brown adipose tissue (BAT) RNA was extracted and reverse-transcribed into cDNA for real-time PCR quantification of *Grk5*normalized to *18s* (endogenous control). **(C)** Six-week-old male C57BL/6J mice were fed chow or a high fat diet (Research Diets Inc #D12492, 60% from fat) for 12 weeks and their body composition such as fat mass was quantified by EcoMRI (n=10/diet group). **(D)** Adipocyte fraction and stromal vascular (SV) cell fraction were isolated from the Epi Vis WAT of overnight fasted mice. Both fractions’ RNA was extracted and reverse-transcribed into cDNA for real-time PCR quantification of *Grk5*normalized to *18s* (endogenous control). All results are mean ± SEM, presented as the fold change compared to chow-fed mouse group and analyzed using a two-tailed Student’s unpaired t-test **(A-C)**, or the fold change compared to chow SV fractions and analyzed using a one-way ANOVA with Sidak multiple comparisons **(D)**.

**Figure 2:**
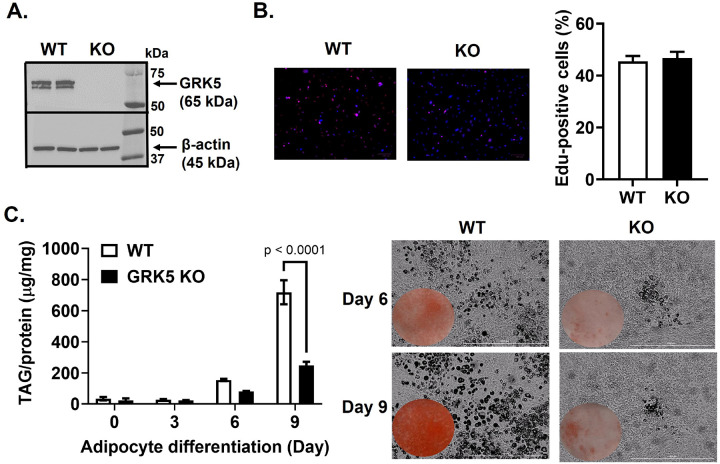
The effect of GRK5 deficiency on adipocyte differentiation. **(A)**Cellular proteins of undifferentiated wildtype (WT) control and GRK5 knockout (KO) 3T3-L1 pre-adipocytes (n=2/genotype) were harvested and subjected to Western blot using anti-GRK5 and anti-β-actin antibodies. **(B)** After 2 days of growth, proliferation was assessed in undifferentiated WT and GRK5 KO 3T3-L1 pre-adipocytes (n=6/genotype). The percentage of EdU-positive cells (pink) was calculated by merging EdU (red) and Hoechst 3342 (blue) staining. **(C)** WT and GRK5 KO 3T3-L1 cells (n=3/genotype) were proliferated for 2 days (Day 0) and then differentiated into adipocytes for 9 days. Day 0, 3, 6, and 9 cells were lipid-extracted to measure triacylglycerol (TAG) mass by a colorimetric assay. Daily Cytation images at 10x magnification were taken during 9 days of adipocyte differentiation. All results are mean ± SEM and analyzed using a two-tailed Student’s unpaired *t* test **(B)**and a two-way ANOVA with Sidak multiple comparisons **(C)**.

In support of previous work showing a positive correlation between GRK5 gene expression and adipose tissue mass in rats (4) and humans (5), we demonstrate here that high fat diet-induced obese mice versus chow-fed lean mice (Fig. 1A) displayed ~ 2-fold increased *Grk5* mRNA levels in white epidydimal visceral white adipose tissue, but not in brown fat (Fig. 1B). We further found that *Grk5* mRNA is more abundant in stromal vascular (SV) fractions than in adipocyte fractions isolated from the epidydimal visceral white adipose tissues of both chow-fed lean and a high fat diet-induced obese mouse (Fig. 1C–D). The SV fractions contains stem cells, adipose progenitor cells (committed pre-adipocytes), endothelial cells, and immune cells. Thus, a GRK5 KO 3T3-L1 pre-adipocyte cell line was generated using CRISPR-Cas9 gene editing (**Supplementary Fig. 1A**), confirmed by the Western blot (Fig. 2A), to investigate GRK5 function in adipocyte differentiation.

### GRK5 deficiency impairs adipocyte differentiation.

**Figure 3:**
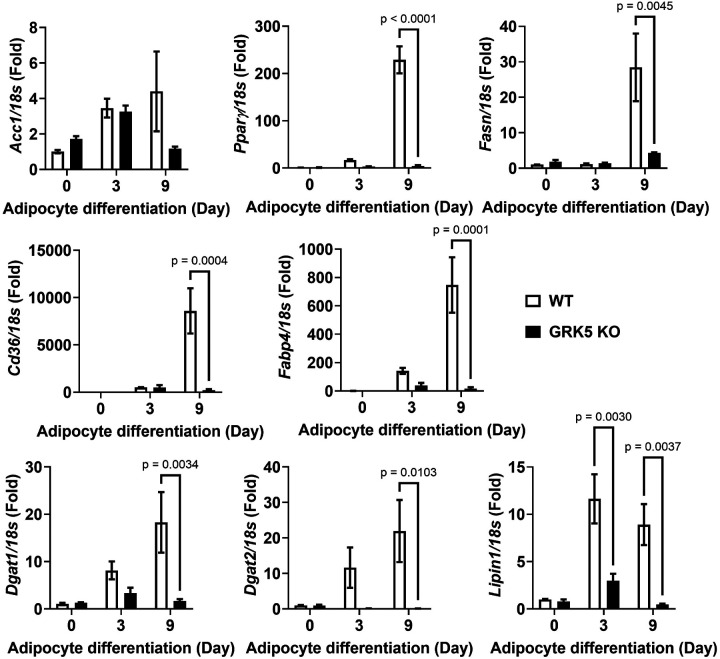
The effect of GRK5 deficiency on adipogenic and lipogenic gene expression. Cellular RNA was extracted from wildtype (WT) control and GRK5 knockout (KO) 3T3-L1 cells (n = 3/genotype) and reverse-transcribed into cDNA for real-time PCR quantification of *Acc1, Pparγ, Fasn, Cd36, Fabp4, Dgat1, Dgat2,* and *Lipin1* normalized to *18s* (endogenous control). All results are mean ± SEM and presented as the fold change compared to WT at Day 0 and analyzed using a two-way ANOVA with Sidak multiple comparisons.

First, we showed that WT and GRK5 KO cells proliferated similarly (Fig. 2B). We then found that, compared to WT, GRK5 KO cells were unable to accumulate TAG and develop into mature adipocytes when exposed to adipogenic stimuli (Fig. 2C). We then examined several key genes that play a role in adipogenesis and lipid metabolism. Except *Acc1*, the mRNA levels of *Pparγ, Fasn, Fabp4, Cd36, Dgat1, Dgat2*, and *Lipin1* were decreased in GRK5 KO compared to WT adipocytes during Day 3–9 of differentiation (Fig. 3). These data suggest that GRK5 deletion in 3T3-L1 cells suppresses adipogenesis and lipid accumulation.

### GRK5 deficiency downregulates IGF-1 signaling and decreases ERK activation.

**Figure 4:**
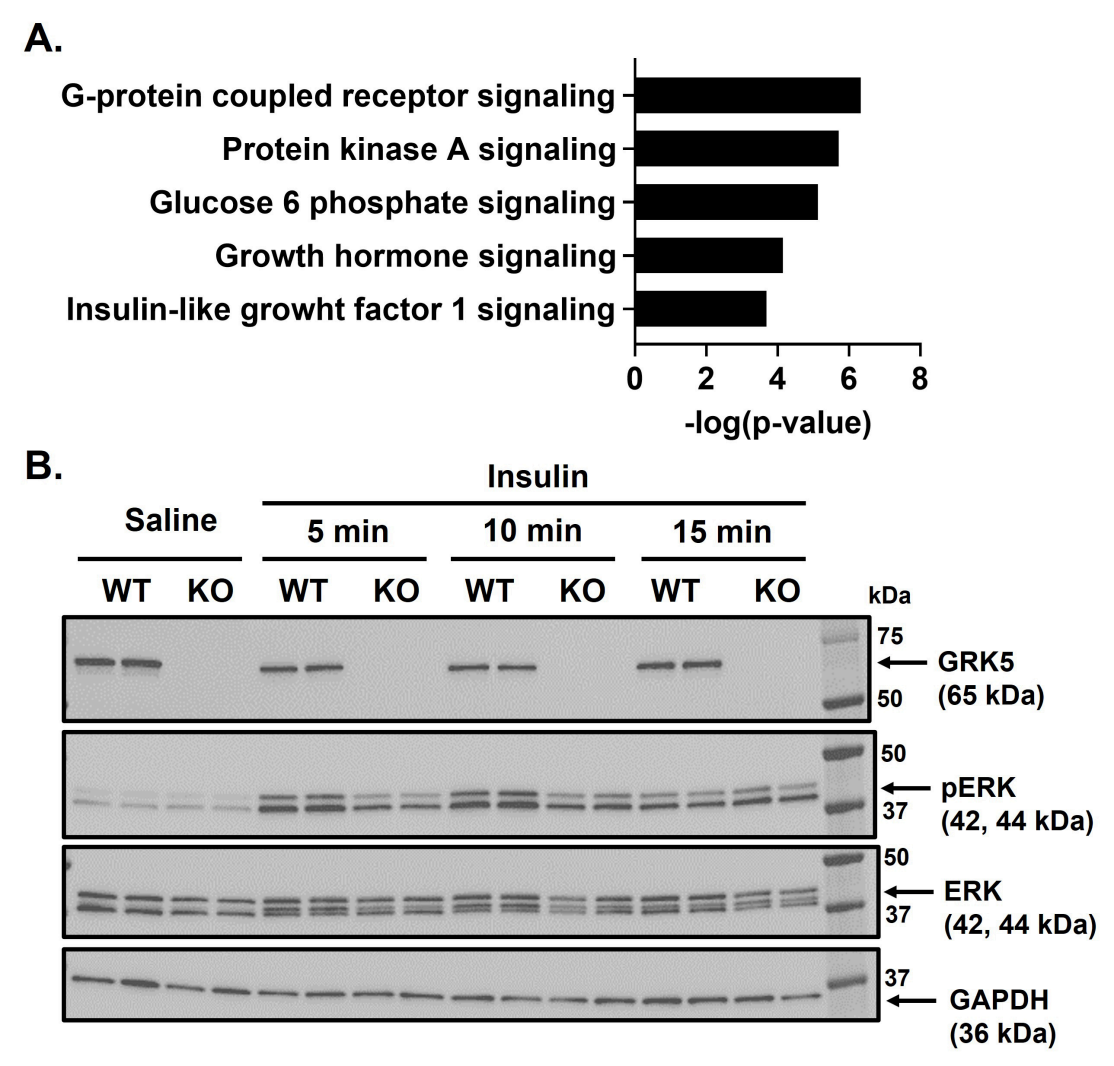
The potential underlying mechanisms of GRK5 in adipocyte differentiation. **(A)** Pathway analysis of RNA sequencing data using Limma. **(B)** Cellular proteins of wildtype (WT) control and GRK5 knockout (KO) 3T3-L1 pre-adipocytes (n=2/genotype) were harvested and subjected to Western blot using anti-GRK5, anti-phosphorylated (p)-ERK, anti-ERK, and anti-GAPDH antibodies.

Next, we applied an unbiased RNA sequencing approach to identify differences in gene signatures between GRK5 KO and WT 3T3-L1 pre-adipocytes. We revealed 164 upregulated and 397 downregulated genes in GRK5 KO relative to WT pre-adipocyte cultures. Based on IPA pathway analysis datasets (**Supplementary Table 1**), we summarized the top 5 most dysregulated pathways involved in adipocyte differentiation (Fig. 4A). As expected, GPCR signaling (p = 4.677×10^− 7^) and protein kinase A (PKA) signaling (p = 1.9498×10^− 6^) were the top two significantly dysregulated pathways (Fig. 4A). GRK5 KO cells also have significantly dysregulated IGF-1 signaling (p = 2.04×10^− 4^). GRKs are known signaling molecules shared between IGF-1R, a receptor tyrosine kinase, and GPCRs (24, 25).

Since ERK is an important downstream target for adipogenic insulin-activated IGF-1 receptor signaling for initiating adipogenesis, we examined whether ERK phosphorylation was altered in GRK5 KO versus WT 3T3-L1 cell cultures treated with or without insulin. We found that GRK5 deletion in 3T3-L1 pre-adipocytes resulted in decreased ERK phosphorylation when compared to WT cells after insulin stimulation for 5 minutes (Fig. 4B; Quantification data in **Supplementary Fig. 1B**), indicating that GRK5 may regulate adipocyte differentiation through insulin/IGF-1 receptor and ERK pathways.

### GRK5 inhibitor reduces adipocyte differentiation.

**Figure 5:**
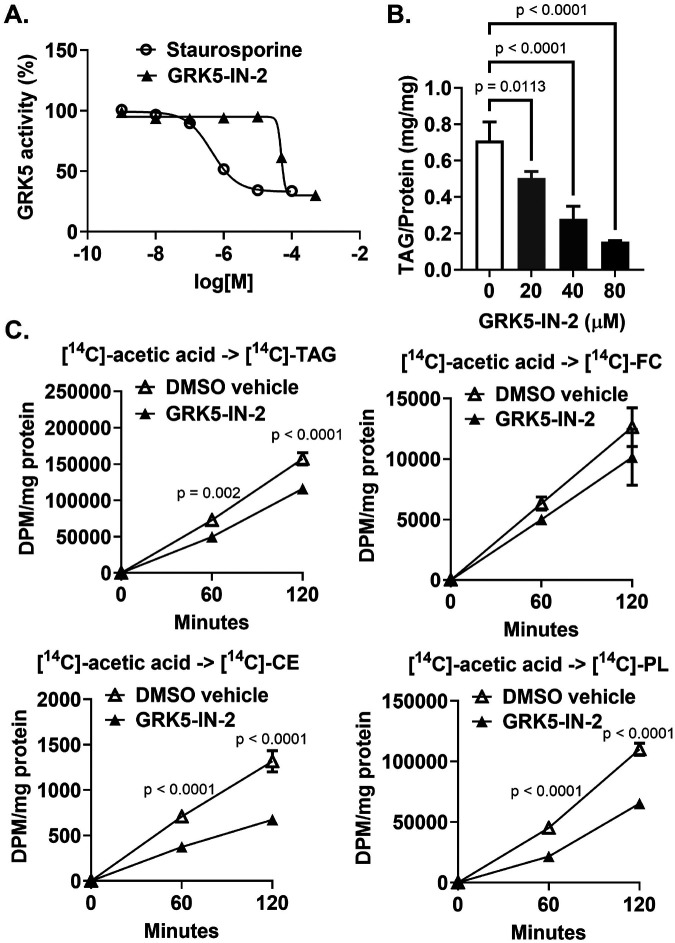
The effect of a GRK5 inhibitor on adipocyte differentiation. **(A)** The dose-response curves of GRK5-IN-2 and staurosporine were determined by a GRK5 kinase system and a luminescent ADP detection assay. **(B)** Wildtype 3T3-L1 pre-adipocytes were differentiated and concurrently treated without or with GRK5-IN-2 (n=3/dose) for 7 days. Cells were lipid-extracted to measure triacylglycerol (TAG) mass by an enzymatic colorimetric assay. **(C)** Day 3 differentiated wildtype 3T3-L1 pre-adipocyte cultures were pre-treated without or with GRK5-IN-2 for 30 minutes and then treated with insulin plus 0.5 μCi/ml of [1,2-^14^C]-acetic acid for 60 and 120 minutes (n=3/time point). Cells were lipid-extracted, and TAG, free cholesterol (FC), cholesteryl ester (CE), and phospholipid (PL) were separated using thin layer chromatography. [^14^C]-TAG, [^14^C]-FC, [^14^C]-CE, and [^14^C]-PL were quantified by liquid scintillation counting. All results are mean ± SEM and analyzed using a two-way ANOVA with Sidak multiple comparisons.

Because genetic deletion of GRK5 is not a viable therapeutic option, we identified a small molecule GRK5 inhibitor, GRK5-IN-2, and assessed its effect on adipocyte differentiation. Using a GRK5 kinase system and a luminescent ADP detection assay, we found that GRK5-IN-2 had a half maximal inhibitory concentration (IC50) of 49.7 μM as compared to staurosporine, the reference compound with an IC50 of 0.4 μM (Fig. 5A). We found that GRK5-IN-2 treatment significantly decreased TAG synthesis during 7 days of WT 3T3-L1 adipocyte differentiation in a dose-dependent manner (Fig. 5B). Next, we performed functional characterization. Day 3 differentiated WT pre-adipocyte cultures were treated with [^14^C]-acetic acid to determine de novo lipogenesis or [^3^H]-oleic acid to measure fatty acid uptake and esterification. GRK5-IN-2 inhibitor treatment significantly decreased the rate of TAG, cholesteryl ester (CE), and phospholipid (PL), but not free cholesterol (FC), synthesis from [^14^C]-acetic acid (Fig. 5C). However, GRK5 inhibition did not affect [^3^H]-oleic acid uptake (**Supplementary Fig. 2B**) as well as [^3^H]-TAG and [^3^H]-PL formation from [^3^H]-oleic acid (**Supplementary Fig. 2C**). These data suggest that the effect of GRK5 inhibition by GRK5-IN-2 is on adipogenesis and insulin-stimulated *de novo* lipogenesis, not on fatty acid uptake and esterification into lipids.

## Discussion

In the current study we show that *Grk5* is expressed in the stromal vascular fraction of white adipose tissue in mice, and that GRK5 KO pre-adipocytes fail to differentiate into mature adipocytes, possibly as a result of impaired insulin-stimulated IGF-1 receptor and ERK activation. We further demonstrate that a novel GRK5 inhibitor, GRK5-IN-2, leads to decreased insulin-stimulated *de novo* lipogenesis. Together, these results emphasize the importance of GRK5 in adipocyte differentiation, suggest a mechanistic pathway for its action, and identify a novel inhibitor that may have therapeutic promise.

The current work supports previous findings (4, 5) by showing that diet-induced obese mice versus chow-fed lean mice had increased GRK5 mRNA levels (~ 2 fold) in white, but not in brown adipose tissue (Fig. 1A–B). Similarly, Wang et al. found that GRK5 mRNA levels were significantly elevated in white (~ 2 fold), but not in brown, adipose tissue in male C57BL/6J mice fed a high fat diet compared with mice fed a standard lab diet (6). They also reported that whole body GRK5 KO mice exhibited protection from diet-induced obesity (6). We show here that *Grk5* mRNA is abundant in the stromal vascular fraction, but not adipocyte fraction, of mouse white adipose tissue (Fig. 1C), supporting previous findings (10, 11). Therefore, we created a novel GRK5 KO 3T3-L1 pre-adipocyte to study the mechanical role of GRK5 in adipocyte differentiation.

During adipogenic stimuli, GRK5 KO 3T3-L1 pre-adipocytes acquired adipocyte morphology slower and accumulated less TAG than WT control cells (Fig. 2), potentially by suppressing expression of several adipogenic and lipogenic genes (Fig. 3). In support of our data, Wang et al. observed that adipogenic gene expression (e.g., *Pparγ, Fasn*, and *Fabp4*) is decreased in white adipose tissue of whole body GRK5 KO mice compared to their littermate control mice on a high fat diet (6). However, the mechanisms by which GRK5 regulates adipogenesis in pre-adipocytes are unknown. Therefore, we performed RNA sequencing and pathway analysis and found that IGF-1 signaling may be a potential underlying mechanism (Fig. 4A).

Canonically, GRKs terminate GPCR signaling though phosphorylation of cognate receptors, resulting in recruitment of β-arrestin, which then facilitates receptor internalization and degradation (7). As a receptor tyrosine kinase, IGF-1 receptor has recently been found to “borrow” molecular components from GPCR signaling, such as GRKs and β-arrestins (24, 25). For example, Zheng et al. reported that GRK2 and GRK6 can physically bind to IGF-1 receptor and phosphorylate IGF-1 receptor at serines 1248 and 1291. These serine phosphorylated binding sites on IGF-1 receptor promoted β-arrestin1 recruitment(26). However, there was an opposing effect of GRK2 and GRK6: knockdown of GRK2 increased whereas knockdown of GRK6 decreased IGF-1 receptor degradation and β-arrestin1-mediated ERK activation(26). Because GRK5 and GRK6 belong to the same subfamily(7), we hypothesized and demonstrated that in a similar manner to GRK6 knockdown, GRK5 deficiency reduced ERK activation in insulin-treated 3T3-L1 pre-adipocyte cultures (Fig. 4B). Pre-adipocytes express primarily IGF-1 receptors rather than insulin receptors, so insulin in an adipogenic cocktail promotes adipocyte differentiation by activating IGF-1 receptor and its downstream ERK pathways (18, 27). Future studies are needed to investigate whether GRK5 can bind and phosphorylate insulin-stimulated IGF-1 receptor which will then activate ERK pathway in pre-adipocytes to promote adipogenesis.

Since genetic deletion is not a viable therapeutic option, we have identified GRK5-IN-2, a pyridine-based bicyclic compound (28), as a commercially available small molecule GRK5 inhibitor. Our cell data demonstrated that GRK5-IN-2 treatment resulted in decreased adipogenesis much like our GRK5 KO cell line, as well as decreased *de novo* lipogenesis (Fig. 5), suggesting that GRK5 inhibition may be a therapeutic target for treating and preventing obesity. Although there are other GRK5 inhibitors such as KR-39038 (29), we chose GRK5-IN-2 because synthesizing KR-39038 is very time-consuming and expensive, which may limit its use in clinical settings. We are currently conducting preclinical studies using GRK5-IN-2 to see if we can replicate our cell results in a diet-induced obese mouse model and to determine whether GRK5 inhibition could be a valuable translation target.

## Data Availability

All the data generated during and/or analyzed during the current study are available from the corresponding author on reasonable request.
